# Macrophage TLR4 and PAR2 Signaling: Role in Regulating Vascular Inflammatory Injury and Repair

**DOI:** 10.3389/fimmu.2020.02091

**Published:** 2020-09-18

**Authors:** Sheikh Rayees, Ian Rochford, Jagdish Chandra Joshi, Bhagwati Joshi, Somenath Banerjee, Dolly Mehta

**Affiliations:** Department of Pharmacology and Center for Lung and Vascular Biology, College of Medicine, University of Illinois, Chicago, IL, United States

**Keywords:** macrophage, vascular permeability, acute lung injury, TLR4, PAR2, inflammation, alveolar macrophages

## Abstract

Macrophages play a central role in dictating the tissue response to infection and orchestrating subsequent repair of the damage. In this context, macrophages residing in the lungs continuously sense and discriminate among a wide range of insults to initiate the immune responses important to host-defense. Inflammatory tissue injury also leads to activation of proteases, and thereby the coagulation pathway, to optimize injury and repair post-infection. However, long-lasting inflammatory triggers from macrophages can impair the lung's ability to recover from severe injury, leading to increased lung vascular permeability and neutrophilic injury, hallmarks of Acute Lung Injury (ALI). In this review, we discuss the roles of toll-like receptor 4 (TLR4) and protease activating receptor 2 (PAR2) expressed on the macrophage cell-surface in regulating lung vascular inflammatory signaling.

## Introduction

Macrophages (MΦ), initially classified as phagocytes by Metchnikoff in 1893 (1, 2), constitute a heterogeneous group of phenotypically and genetically distinct immune cells located within the lungs (3–9). Lung MΦ demonstrate high expression of pathogen recognition receptors (PRRs), such as Toll-like receptor 4, and play a dual role: initially, they trigger inflammatory signaling (10), but later signal removal of cellular debris and restoration of tissue homeostasis (11–13). Long lasting inflammatory signaling can impair the tissue repair process, leading to development of Acute Lung Injury (ALI). ALI frequently develops following sepsis, trauma or pneumonia, and if unresolved, may progress to Acute Respiratory Distress Syndrome (ARDS), resulting in high mortality and morbidity (14–18).

Alveolar macrophages (AMΦ) and interstitial macrophages (IMΦ) constitute the two key resident MΦ populations in the lungs. AMΦ, as the name suggests, are located within the airspace of the alveoli, juxtaposed to epithelial cells (19). Interstitial macrophages (IMΦ), on the other hand, have a more varied localization and have been shown to lie in the bronchi, airways, and interalveolar space shared by fibroblasts and other mesenchymal cells (5, 6, 20). A few studies have identified intravascular MΦ as a third resident population in the lung, but their existence remains questionable (21). Additionally, monocytes recruited to inflamed tissue differentiate into tissue macrophages (22). Macrophages can also “polarize” along a continuum between two states designated M1 (pro-inflammatory) and M2 (anti-inflammatory) in response to different cytokines and tissue environments (23–25). However, the mechanism by which AMΦ, IMΦ, or recruited macrophages acquire pro-inflammatory or anti-inflammatory lineages and the signaling involved in their transition to these lineages during injury remains a topic of fierce debate.

Inflammation is also known to activate the coagulation cascade, which in turn affects inflammatory processes by generating a further suite of proteases such as trypsin, thrombin, elastases, FVIIa and FXa (26, 27). Protease activated receptors (PAR) such as PAR2, are known to ligate trypsin, tryptase, factor VIIa, factor Xa, and elastase (28, 29). Interestingly, recent studies suggest that thrombin also ligates PAR2 (30, 31). How then does PAR2 signaling affect TLR4-mediated inflammatory responses in lung MΦ. In this review, we focus on lung resident MΦ populations and the recently discovered coupling between TLR4 and protease activating receptor 2 (PAR2) signaling in regulating injury repair.

## Lung Resident Macrophages

**Figure 1:**
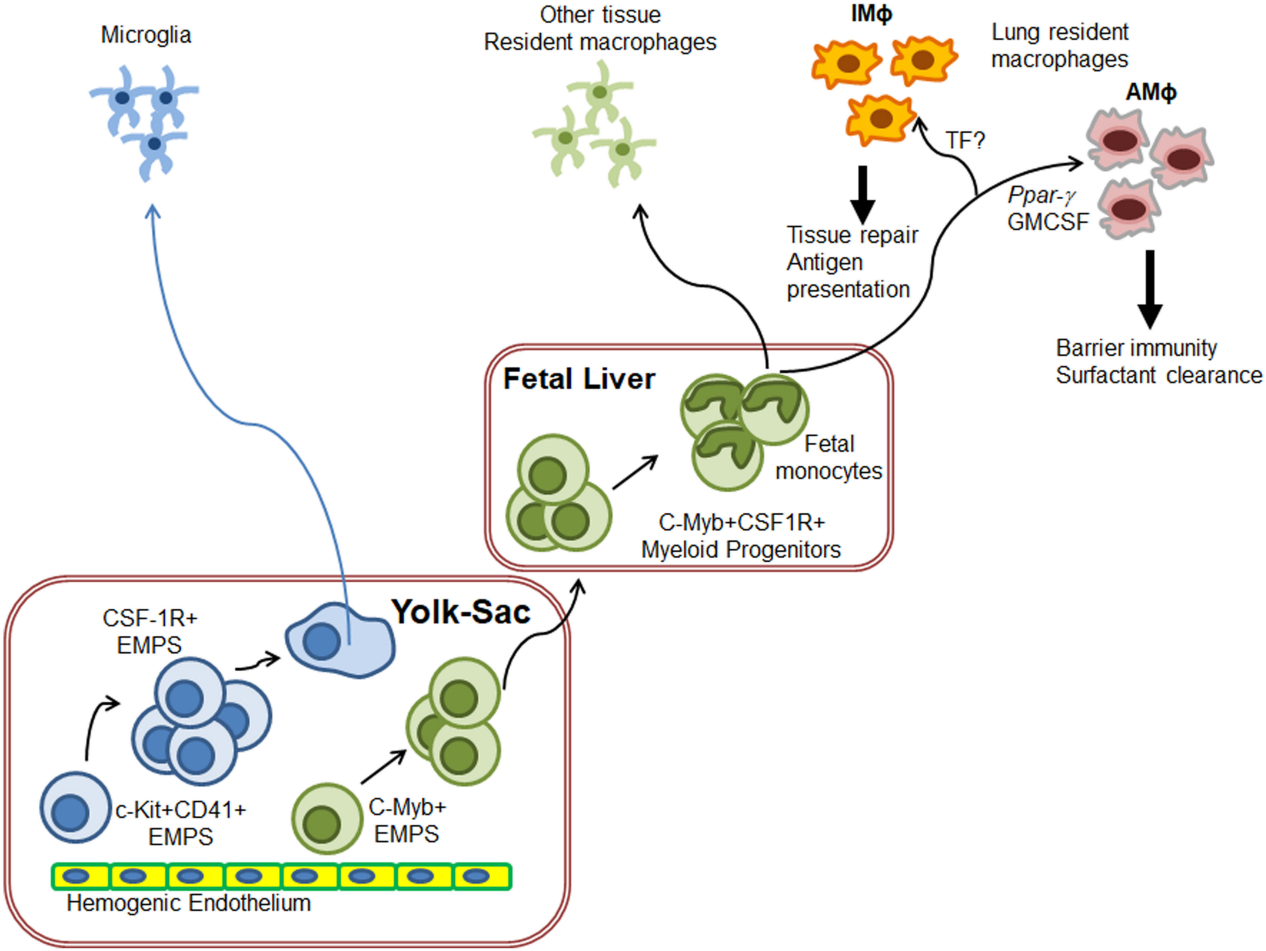
Schematics of generation of lung resident macrophages. Based on the ontogeny of tissue resident macrophages (TRM), microglia originate directly from yolk-sac (YS) macrophages while other TRM originate from fetal liver monocytes. In the case of lungs, F4/80^+^ embryonic YS-MΦ seed the budding lung around E10.5 as primitive IMΦ. On E12, fetal liver monocytes enter the alveoli after birth and differentiate into AMΦ to regulate lung surfactant generation and host response F4/80^+^ bone marrow MΦ also arrive at the lung on E16 where they expand to form “definitive” IMΦ. IMΦ role needs to be defined but these are predicted to induce wound repair. Exact molecular control of IMΦ generation has not yet been fully deciphered.

Investigations into the ontogeny of the AMΦ and IMΦ populations have uncovered very distinct origins during their development (Figure 1). The Kosnav lab investigated the developmental origin of lung MΦ and showed that embryonic MΦ colonize the lung in three successive waves (32). In the first wave, F4/80^+^ embryonic MΦ from the yolk sac migrate into the lung bud around E10.5. These MΦ persist in the adult lung as “primitive interstitial MΦ” and localize peripherally and perivascularly. The second wave is initiated by Mac2^+^ embryonic monocytes at E12, most likely from the fetal liver (33), which enter the alveoli after birth and differentiate into AMΦ. The third wave, made up of F4/80^+^ bone marrow MΦ, arrives at the lung on E16 and expands to form “definitive” interstitial MΦ. Both F4/80 lineages cease to express F4/80 and begin expressing MHCII during the first 3 weeks of postnatal life. In humans, AMΦ can be detected in full term healthy infants as well as all infants who survive for 48 h after birth, irrespective of health (34). However, a study showed that AMΦ could be detected in a 20-week human fetus with congenital pneumonia (34), indicating that the lung niche may drive AMΦ generation prenatally.

The luminal surface area of adult human lungs ranges from 50 to 100 m^2^ (35), larger than any other soft tissue, including the skin (2 m^2^) (36) or the gut (10 m^2^) (37). Because of their localization in the pulmonary epithelium, AMΦ are directly exposed to the external environment and so are the first immune cells to react to inhaled pathogens and pollutants. Additionally, AMΦ maintain the surfactant layer which prevents collapse of alveoli during respiration (38–40). On average, there is a single AMΦ for every three alveoli in mice (41). In humans, AMΦ constitute about ~3–5% of all cells in a healthy lung (42). These AMΦ can be sessile or motile in nature. Westphalen et al. demonstrated that sessile AMΦ communicate directly with the alveolar epithelium to dampen immune responses (41). However, Paeo et al. described an AMΦ population that seems to move to-and-fro between alveoli through interalveolar fenestrae, the so-called Kohn pores (43). It is recognized that monocytes can also access the alveolar space and differentiate into AMΦ over the course of pulmonary disease, such as pulmonary fibrosis (44, 45). But whether this occurs during acute lung injury remains to be established.

IMΦ, initially referred to as septal cells (46), comprise a relatively small population of lung MΦ, ranging from a tenth to a half of the total number of AMΦ (8, 47–51). Many studies have defined IMΦ as precursors of AMΦ (44, 52). IMΦ contribute to tissue remodeling and maintenance as well as antigen presentation and thereby influence dendritic cell functions (38, 53–57). However, IMΦ have less phagocytic potential when compared to AMΦ (58).

Both AMΦ and IMΦ express conventional macrophage markers, such as CD64, CD68, MAC2, CD11b, CD11c, MERTK (59). Phenotypically, AMΦ are clearly separated from IMΦ and other non-alveolar MΦ through cell-surface expression of Itgax (CD11c), and Siglec 5 (Siglec F) (60) but lack Itgam (CD11b) expression. IMΦ can also be discriminated visually from AMΦ by their smaller size and smoother surface. Surface markers that specifically identify IMΦ remain to be established (61). However, CD11b, CX3CR1, MHCII, CD11c without SiglecF have all been used to identify IMΦ and other non-alveolar MΦ (6, 62). Recently, attempts have been made to categorize IMΦ into phenotypic and anatomical subsets such as Lyve1^hi^ and MHCII^lo^ IMΦ residing near blood vessels and Lyve1^lo^ MHCII^hi^ IMΦ residing near nerve fibers or endings. One study has suggested that Lyve1^hi^ IMΦ are responsible for exacerbated fibrosis and that both IMΦ populations are slowly replaced by Ly6C^hi^ monocytes over time (5). However, this notion is debated given that different subsets of monocytes are known to exist in adult non-diseased human lungs and naïve mice (62–68).

Transcriptional profiling of AMΦ indicated that GM-CSF secretion from alveolar epithelial type-II cells (ATII cells) along with MΦ-peroxisome proliferator-activated receptor γ (PPARγ) is required for differentiation and maintenance of the AMΦ phenotype from embryonic precursors (33, 69). This mechanism seems to be conserved in mice (33, 39, 69–71) and humans (72–75). Recent studies suggest that autocrine TGF-β signaling is also essential to maintain AMΦ lineage (76). Additionally, basophil imprinting of cytokines, such as IL-33 and GM-CSF (77), as well as L-plastin, an actin binding protein, were shown to contribute in generating AMΦ from fetal monocytes (78, 79). Transcription factors Bach1 and Bach2 have been shown to be involved in regulating AMΦ maintenance of lung surfactant homeostasis (80, 81). Moreover, once differentiated, resident AMΦ also self-proliferate to maintain their lineage (82), although it has been demonstrated that circulating monocytes contribute to this pool by differentiating into AMΦ following tissue injury or infection (83). Future studies will unravel additional transcriptional and signaling mechanisms by which monocytes, IMΦ or AMΦ themselves maintain the AMΦ pool during injury. Compared to AMΦ, transcriptional regulation of IMΦ is still in its infancy.

## Macrophage Toll-Like Receptor 4 and Signaling

Pattern or pathogen recognition receptors (PRRs) are a class of receptors that recognize pathogen-associated molecular patterns, PAMPs, of pathogenic organisms or endogenous signals from damaged cells, referred to as damage-associated molecular pattern or DAMPs. Upon binding with PAMPs or DAMPs, PRRs activate signaling cascades that lead to the production of pro-inflammatory cytokines and interferons, an important step in the initiation of adaptive immunity (84–86). Endocytic or phagocytic PRRs, such as mannose receptors, can aid in the recognition and intake of microbes by MΦ (87, 88).

TLRs contain 22–29 residue long leucine-rich repeats- (LRR-) N-terminal ectodomains and intracellular toll-interleukin-1 receptor (TIR) signaling domains. The LRR motif of TLRs play a key role in the protein-protein interactions involved in downstream signaling (89). MΦ have around 10 TLRs in humans and 13 in mice. Out of these, TLR 1, 2, 4, 5, and 6 are located on the cell membrane, while TLR 3, 7, 8, and 9 are intracellular (90–92). The TLR family recognizes a diverse range of DAMPs or PAMPs, such as lipoproteins, di- and triacyl lipopeptides, lipoteichoic acid, peptidoglycan, fungal zymosan, double-stranded RNA, flagellin, unmethylated CpG DNA, and LPS. A component of the cell wall from gram-negative bacteria, LPS, contains lipid A, a non-repeating “core” of oligosaccharide, and a distal polysaccharide. Lipid A has the endotoxic properties recognized by TLR4 (93, 94) and is a typical PAMP used in studies centered on TLR4 signaling.

TLR4 is unique among the various TLRs due to its ability to activate signaling from the cell-surface as well as intracellularly. Cell-surface TLR4 propagates signaling through both a MyD88-dependent and independent pathway, resulting in generation of proinflammatory cytokines and type I interferons, respectively (95, 96). Upon binding LPS, cell-surface TLR4 recruits several adaptor proteins through its intracellular TIR domain (97). These adaptor proteins include MyD88, TRIF, MyD88 adapter-like (Mal/TIRAP), sterile and armadillo motif-containing protein (SARM), TRIF-related adaptor molecule (TRAM), tumor necrosis factor receptor associated factor6 (TRAF6) and the serine-threonine kinase, IL-1R-associated kinase (IRAK). TLR4 immune signaling is further accelerated by accessory molecules such as CD14, CD36, and myeloid differentiation2 (MD2) (98). TLR4-MyD88 signaling is mediated through complex formation between MyD88, phosphorylated IRAK, and TRAF6 which in turn activates the transcription factor, NFκB and MAPK to induce the generation of several pro-inflammatory cytokines, such as TNF-α and IL-6 (99, 100). In contrast, MyD88 independent TRIF-mediated TLR4 signaling occurs through activation of transcription factor, IFN regulatory factor 3 (IRF3) and STAT1, which leads to generation of IFN-β, IL-10, and RANTES, as well as late phase NFκB activation (100, 101). Both of these pathways propogate at the plasma membrane simultaneously, but recent studies suggest that TRAM-TRIF signaling can also be initiated following endocytosis of TLR4 (101, 102).

Endocytosed TLR4 can sense cytosolic LPS to induce NFκB and IRF3 mediated transcription, which is critical to full regulation of innate immunity during pathogenic insult (100, 102). Studies show that p120-catenin (p120), a member of a subfamily of armadillo repeat domain containing proteins, promotes the endocytosis of TLR4 in MΦ and stimulates TRIF, which in turn activates the transcription factor IRF3 to enhance the expression of type 1 interferons (92, 100).

Additionally, TLR4 activates the formation of inflammasomes, also known as inflammatory signaling platforms, by inducing the cytosolic innate immune sensor NLRP3, adaptor apoptosis-associated speck-like protein containing a caspase recruitment domain (ASC) and caspase-1 (103–105). Inflammasome activity requires both priming by TLR4-NFκB mediated production of pro-IL-1β and pro-IL-18 and an NLRP3-specific signal activated by either reactive oxygen species (ROS), extracellular ATP, alum, or pore-forming toxin nigericin. Upon activation, NLRP3 and ASC form a complex with pro-caspase-1 through homotypic domain interaction, leading to generation of active caspase-1, which cleaves pro-IL-1β and pro-IL-18 to the mature IL-1β and IL-18 forms. Evidence suggests that efflux of K^+^ across the plasma membrane is a key factor regulating the activation of NLRP3. Di et al. recently showed that NLRP3 activation of K^+^ efflux by two-pore domain weak inwardly rectifying K^+^ channel 2 (TWIK2) played a critical role in regulating inflammasome formation in AMΦ (106).

Recent studies show that, in mice, caspase-11 (caspases-4 and 5 in humans) can bind cytosolic LPS and induces the NLRP3 pathway as well as gesdermin D to stimulate pyroptosis (105, 107) and the release of IL-1β. While pyroptosis, defined as gasdermin-mediated regulated necrosis, protects organisms from invading pathogens, it may cause local as well as systemic inflammation, including septic shock (108, 109).

Cell death leads to the generation of reactive species and activation of Z-DNA binding protein 1 (ZBP1). ZBP1 results in the release of mtDNA and/or dsDNA. Cyclic GMP-AMP synthase (cGAS) catalyzes generation of cyclic GMP-AMP (cGAMP) upon binding to dsDNA, which leads to the activation of STING/IFN-β signaling and lung injury (108, 110–112). Stimulator of interferon genes (STING), a transmembrane homodimer located in the endoplasmic reticulum (ER) membrane, has recently emerged as a potent inducer of MΦ inflammatory signaling following tissue injury (112). Joshi et al. recently showed that recruited MΦ were required to dampen AMΦ-STING signaling. They demonstrated that ER-localized sphingosine kinase-2 (SPHK2) generated sphingosine-1-phosphate (S1P), which prevented cGAMP activation of STING and thus attenuated lung vascular injury. Oxidized PAPC (oxPAPC) formed from phospholipid, 1-palmitoyl-2-arachidonoyl-sn-glycero-3-phosphorylcholine (PAPC) and lipoproteins (113, 114) also modulated TLR4-induced inflammatory responses. At a very low concentration, oxPAPC antagonized TLR4-induced inflammation and injury, yet at higher doses enhanced the proinflammatory response to TLR4 signaling (113). While the exact mechanism of oxPAC anti-inflammatory function remains unclear, it was shown to inhibit NFκB transcription factor activity by generating cAMP (114) or by binding to CD14 and LPS binding protein (LBP), thereby reducing the sensitivity of TLR4 to LPS (114).

## Macrophage Protease Activated Receptors

Inflammation-induced injury releases a mélange of proteases, complements, chemokines, prostaglandins, and other inflammatory molecules, which activate several receptors, including G-protein coupled receptors (GPCRs) (115). Thus, in addition to expressing TLRs to detect pathogens, MΦ also express an array of GPCRs on their cell-surface, whose function is to optimize the inflammatory response and host-defense function (116, 117). Culture conditions, such as GM-CSF vs. M-CSF, seem to dictate the expression of different sets of GPCRs on MΦ (118). However, AMΦ heavily express complement receptors (C5R1; C3AR1), formyl peptidyl receptor 2 (FPRL2) and several chemokine receptors (CXCR6, CCR8, CCR4, CCR5 etc.) (119).

Protease activated receptors (PARs), PAR1, PAR2, and PAR3 encoded by the genes F2R, F2RL1, F2RL2, and F2RL3, respectively are also expressed on the MΦ cell-surface. As the name indicates, PARs are activated by several different proteases, including those involved in the coagulation pathway (26, 28, 120). While each of these receptors can be cleaved by their specific proteases, several common proteases can also cleave various PARs because of their sequence homology. For example, PAR1 is cleaved by proteases such as thrombin, factor Xa, plasmin, MMP1 and MMP13 (121). Originally, PAR2 was thought to be cleaved only by trypsin, tryptase, factor VIIa, factor Xa and elastase (28, 29). However, recent studies show that thrombin can also cleave PAR2, albeit at higher concentrations (30, 31). PAR3 is cleaved by thrombin only. These proteases cleave PARs at defined sites within the N-terminus, unmasking new N-terminal peptides as tethered ligands. The tethered ligand then binds intramolecularly to a conserved second extracellular loop of cleaved receptor to trigger signaling through heterotrimeric G-proteins. However, subtle mechanistic differences exist among these proteases in initiating the activity of the relevant canonical pathway, depending on the PAR in question. Synthetic PAR peptides or activating peptides (APs) mimic the tethered ligand domains. These peptides directly activate their respective PARs, bypassing the proteolysis process (26, 122). Recent findings indicate that activation of PARs, specifically PAR2, expressed on AMΦ suppress TLR4 signaling, as we will discuss further.

## Macrophage PAR2 and Downstream Signaling

PAR2 couples to G_α_s, G_α_q, G_α_i, and G_α_12/13 and triggers several signaling cascades to mediate its diverse cellular functions (31, 123, 124). The canonical activation of PAR2 by its proteases occurs after hydrolysis at the R^36^/S^37^ position. The exposed tethered ligand domain, SLIGRL (mouse) and SLIGKV (human), in turn binds to initiate PAR2 signaling. Other proteases, including thrombin, neutrophil elastase, cathepsin G, cathepsin S, proteinase-3, gingipain-R and kallikrein-14, cleave PAR2 at sites other than the tethered ligand site, leading to bias signaling (26, 122). Non-mammalian proteases such as LepA and elastase EPa, both secreted by *Pseudomonas aeruginosa*, also cleave PAR2 to either activate or deactivate its downstream signaling (125, 126). Activation of PAR2 by *Pseudomonas aeruginosa* has been shown to cause IFN-gamma production as a mechanism for stimulating bacterial clearance. Similarly, gingipain R produced by *Porphyromonas gingivalis*, Pen C secreted by *Penicillium citrinum* and supernatant from *Propionibacterium acnes* cultures can activate PAR2 (120, 122, 127). Additionally, several small molecule agonists of PAR2 have been synthesized, but their therapeutic efficacy remains uncertain (26, 128).

Classically, GPCR activation is followed by desensitization. GPCR phosphorylation uncouples it from its cognate G-protein and induces its binding to β-arrestin (129), facilitating receptor internalization by recruiting endocytic proteins (130, 131). PAR2 activation is associated with phosphorylation of its cytoplasmic tail, which is responsible for desensitization or internalization of PAR2 (132). The serine and threonine residues within the cytoplasmic tail of the receptor and third intracellular loop are the prime phosphorylation sites; however, it may occur at tyrosine residues as well (133). Ricks and Trejo showed that, compared to wild-type PAR2, desensitization was considerably reduced in PAR2 mutants in which all serine and threonine phosphorylation sites in the C-terminal tail were mutated to alanine (132). Moreover, wild-type phosphorylated PAR2 was internalized through a canonical dynamin, clathrin- or β-arrestin-dependent pathway, but the PAR2 mutant was internalized through a dynamin-dependent and clathrin- and β-arrestin-independent pathway.

### PAR2 and Calcium Signaling

An increase in cytosolic Ca^2+^ is required for the regulation of several cellular processes (134). Agonist-induced increases in cytosolic Ca^2+^ occur by depletion of endoplasmic reticulum (ER) Ca^2+^ stores, followed by Ca^2+^ entry through plasmalemmal channels (135). PAR2 activation via its cognate agonists, such as trypsin, tryptase or agonist peptide, has been demonstrated to increase cytoplasmic Ca^2+^ levels via the phospholipase C-inositol trisphosphate (PLC-IP_3_) axis (136–138). Ca^2+^ signaling by PAR2 is typically activated via Gαq/G11 and influences several intracellular targets, resembling PAR1 signaling. However, evidence shows that trypsin activation of PAR2 can also induce Ca^2+^ signaling by stimulating Gαi/Gαo (139, 140), indicating that coupling of PAR2 to G proteins may depend on variations in the density of cell-surface PAR2, availability of G proteins, or downstream effector protein interactions.

Transient receptor potential channels (TRP) are a group of Ca^2+^-permeable non-selective cation channels involved in MΦ activation. Studies showed that TRPM2 and TRPV4 but not STIM-mediated store-operated calcium channels play an important role in mediating Ca^2+^ entry in MΦ (31, 106, 141). However, it appears that PAR2 was required to suppress TRPV4-mediated Ca^2+^-entry in AMΦ (31). TRPV4 is a polymodally gated channel involved in several fundamental physiological functions of both sensory and non-sensory cells (142). It is also known to play a significant role in several pathophysiological processes, such as asthma, pulmonary fibrosis, cystic fibrosis, sepsis, and lung injury (143–146). TRPV4 is activated by several stimuli including mechanical stress, thermosensation or by intracellular metabolic products (147–149). Also, phospholipase A2 (PLA2)/arachidonic acid (AA) pathway signaling triggered by cell swelling can also activate TRPV4 (150–152).

Rayees et al. showed that thrombin-induced TRPV4 activity was markedly higher in PAR2-null bone-marrow derived macrophages (BMDM) compared to wild-type BMDM, indicating that PAR2 suppresses TRPV4 activity (31). Also, direct activation of TRPV4 with its agonist (GSK1016790A) (153) enhanced Ca^2+^ entry in PAR2-null BMDM more than in wild-type BMDM (31). Further studies will be required to determine whether thrombin activates TRPV4 in AMΦ by generating PLA2 products, cell shape change/swelling or pressure variation.

### PAR2 and Cyclic Adenosine Monophosphate Generation

Cyclic AMP (cAMP) is a ubiquitous second messenger involved in numerous physiological processes in all domains of life. Adenylyl cyclases (AC) generate cAMP from ATP (154). AC have 10 isoforms, nine of which are transmembrane (tm-AC) and regulated by GPCRs, while the soluble form of adenylyl cyclase (sAC) acts as a bicarbonate/pH sensor (155) and is not regulated by G-proteins or forskolin, a direct activator of AC (29, 156). A family of enzymes called phosphodiesterases (PDEs) catabolize cAMP into AMP. There are 11 known PDEs, of which PDE4, 7, and 8 have a strong affinity for cAMP (157–159). cAMP is known to mediate its effects through three target proteins, protein kinase A (PKA), cyclic nucleotide gated ion channels (CNGs and HCNs) and exchange proteins activated by cAMP (EPACs) (154, 160).

PAR2 is known to induce cAMP generation by coupling to Gαs (161, 162). Interestingly, LPS also induced cAMP in MΦ by generating thrombin and activation of PAR2. Forskolin induced a similar increase in cAMP in both wild-type and PAR2-null BMDM. Further, rolipram, a PDE inhibitor, alone or in combination with thrombin, did not induce any significant increase in intracellular cAMP in wild-type or PAR2-null BMDM, indicating that thrombin ligation of PAR2 is necessary for cAMP generation (31). Consistent with this finding, the cell permeable cAMP analog 8-Br-cAMP inhibited thrombin-induced Ca^2+^ entry in PAR2-null BMDM (163). Interestingly, 8-Br-cAMP inhibited TRPV4 induction by GSK1016790A. Additionally, cAMP is known to bind NRLP3 directly to dampen inflammasome generation (164), thus raising the possibility that cAMP generated through PAR2 can suppress both TRPV4 activity and inflammasome generation by TLR4. Though the mechanism by which cAMP inhibits TRPV4 is not yet clear, alignment of the TRPV4 sequence with cAMP PBC domain B, which is conserved in well-known cyclic AMP binding proteins, suggested that cAMP may inhibit the channel by binding to it directly (31). Nonetheless, these results identified PAR2 as a key switch in the control of Ca^2+^ entry in AMΦ through the generation of cAMP.

## Interplay Between TLR4 and PAR2 Signaling

### Role in Macrophage Polarization

As mentioned above, MΦ “polarize” into the M1 or M2 state through dynamic changes in cell response and phenotype, giving rise to the notion that the MΦ dichotomy is crucial for coordinating the initiation, progression, and ultimate resolution of inflammatory injury. However, this conclusion is mainly derived from *in-vitro* studies, using, for example, BMDM and RAW cells (165, 166). The M1 state, or “classically activated” MΦ, is considered pro-inflammatory, characterized by propagation of inflammatory signaling through the secretion of cytokines, such as IL-1β, TNF-α or interferons. LPS, a cell wall component of Gram- bacteria, and IFN-γ polarize MΦ to acquire a M1 state through activation of transcription factors, including NFκB, NFAT and STAT1 (121, 167–169). M2, or “alternatively activated MΦ,” are considered anti-inflammatory, as they induce the arrest of inflammatory signaling and initiate wound healing and other regenerative processes (170). IL-4/IL-13 can program MΦ to adopt the M2 state by activating the STAT6 transcription factor. IL4-activated STAT6 can also compete with STAT1 to repress interferon-γ-mediated responses (168), indicating that M1-MΦ can themselves become M2 as inflammatory injury progresses from the acute phase to the resolution phase. However, recent studies suggest that, while M2 may transition to M1, the reverse is not true due to mitochondrial dysfunction induced by reactive oxygen species produced during M1-MΦ polarization (171).

Human monocytes primarily express PAR1, but upon differentiation into macrophages increase expression of PAR2 (172). PAR2 activation alone is able to skew macrophages into either the M1 or M2 phenotype (173–175). Stimulation of BMDM or RAW cells with the small molecule PAR2 agonist, 2-furoyl-LIGRLO-amide trifluoroacetate salt, skewed MΦ into M1-like cells due to activation of the forkhead box protein O1 (FOXO1) (173). Another study showed that PAR2 activating peptide shifted macrophages into the M1 or M2 phenotype depending on culture conditions. These authors showed that the PAR2 activating peptide SLIGKV, skewed GM-CSF-derived peripheral blood monocytes (PBMC)-MΦ into the M1 phenotype, while MCSF-derived PBMC-MΦ were skewed to the M2 phenotype (175). However, conjoint activation of PAR2 and TLR4 in peritoneal MΦ polarized them toward the M2 phenotype, since PAR2 peptide suppressed the LPS-mediated increase in M1 cytokines (TNFα, IL-6 and IL-12p40) (174). Similarly, other studies have shown that PAR2 null primary macrophages secreted less IL-4/IL-13 in response to LPS as compared to wild-type macrophages, and PAR2 activation was associated with greater M2 cytokine expression after LPS exposure (174, 176).

### Role in Regulating Inflammatory Signaling

Inflammatory signaling induces the expression of tissue factor (TF) and elastase in leukocytes and monocytes, which facilitates activation of the coagulation pathway in part through the production of thrombin (31, 177). TF is also constitutively expressed by cells segregated from blood, mostly epithelial cells and macrophages (115, 178). However, a few studies have addressed the role of PAR2 activation by TF, elastase and thrombin in altering the TLR4-induced inflammatory cascade in alveolar macrophages *in vivo* (179–181). Rallabhandi et al. initially demonstrated, using a heterologous system, that TLR4 and PAR2 receptors physically interact, leading to receptor cooperativity and enhancement of pro-inflammatory signaling through NFκB. They showed that PAR2 activation of NFκB signaling occurred in an adaptor dependent manner. In the presence of TLR4, PAR2-activating peptide (PAR2-AP) enhanced NFκB signaling by recruiting MyD88. However, in the absence of TLR4, the PAR2-AP induced NFκB activity by recruiting the TRIF and TRAM adaptor proteins (181). This could be due to the presence of the TIR (Toll/IL-1 receptor/resistance protein) domain in the C-terminus of PAR2 (182). Thus, without TLR4, PAR2 signaled by recruiting TRIF/TRAM to the C-terminus of PAR2, but this interaction was dislodged by MyD88 in the presence of TLR4 (181).

**Figure 2:**
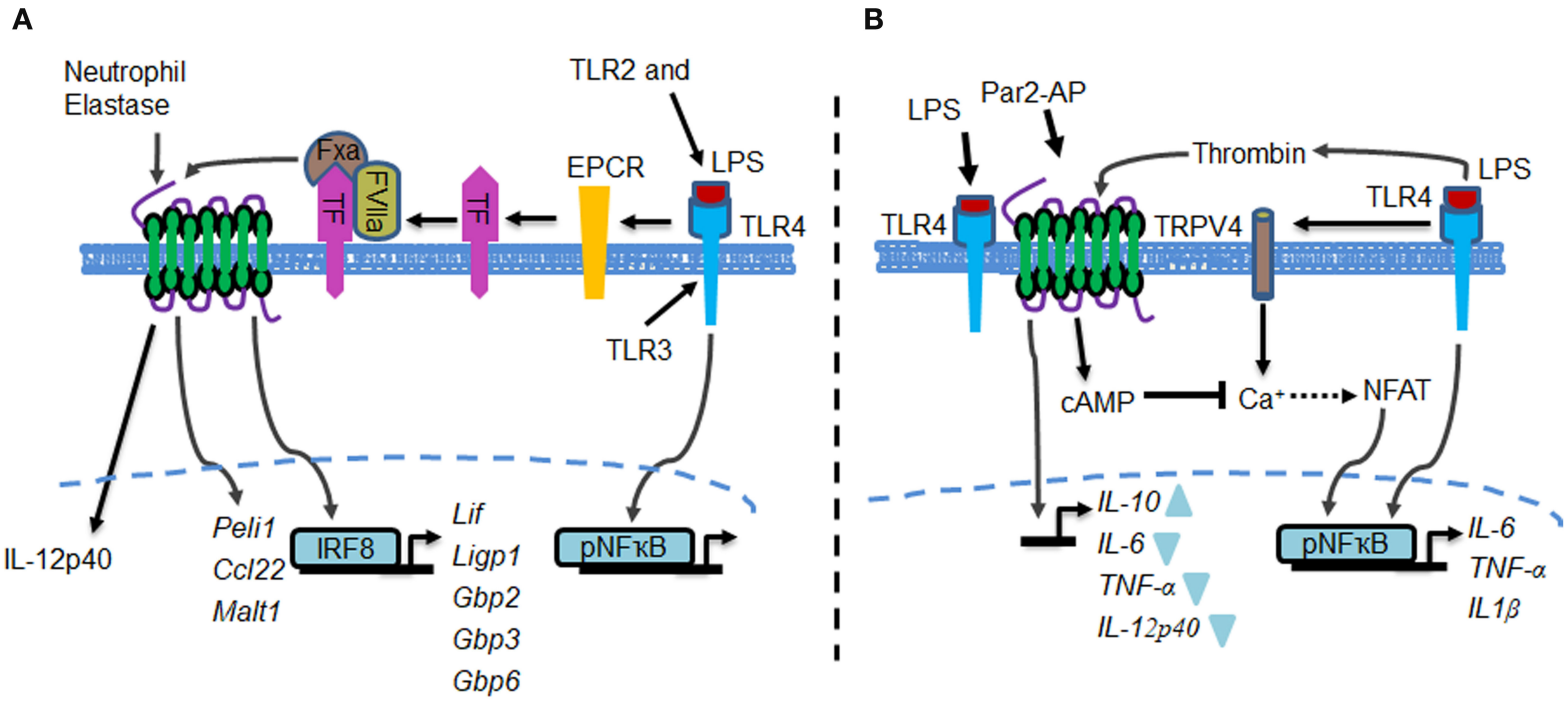
Potential crosstalk models between TLR4 and PAR2. **(A)** PAR2 transactivation via EPCR and neutrophil elastase augments LPS-TLR4 inflammatory signaling. Tissue factor activates PAR2 via EPCR. TLR4-EPCR-mediated activation of PAR2 upregulated the expression of Peli1, Ccl22, and Malt1. Elastase secreted from neutrophils may cleave PAR2 to induce IL-12p40 generation in macrophages. Also, TLR2 and TLR3 may contribute to PAR2 regulation of TLR4 signaling. **(B)** PAR2 suppresses TLR4 inflammatory signaling, thereby facilitating resolution of lung injury. Thrombin secreted during TLR4-induced lung injury directly activates PAR2. PAR2 mediates the generation of cAMP, which suppresses TRPV4-induced Ca^2+^ entry. Deletion of PAR2, hence elimination of cAMP generation, fails to suppress Ca^2+^ entry via TRPV4, leading to protracted NFAT and NFκB activities, resulting in long-lasting inflammatory injury. Additionally, simultaneous activation of PAR2 and TLR4 in peritoneal macrophages enhanced IL-10 expression while the expression of TNF-α, IL-6, and IL-12p40 was decreased.

Liang et al. followed up on the TLR4 and PAR2 receptor co-operativity concept discussed above and showed that TLR4 transactivated PAR2, which then enhanced TLR4 signaling (179) (Figure 2A). In this context, they showed that the endothelial cell protein C receptor (EPCR) serves as a bridge to engage PAR2 with TLR4 and induces pro-inflammatory genes in macrophages (179). Thus, they showed that LPS failed to induce interferon-regulated gene expression in several organs, including lungs, in mice lacking EPCR or PAR2 (179) At a mechanistic level, these authors used BMDM and monocytic RAW265.7 cells to show that LPS upregulated the expression of TF, which was followed by TF-VIIa-Xa complex formation. TF-VIIa-Xa assembly was required for EPCR-mediated activation of PAR2, which resulted in induction of expression of Pellino-1 and IRF8 activity and thereby the full-blown, interferon-regulated, gene expression program (Figure 2A).

Another mechanism of transactivation of PAR2 by TLR4 in GM-CSF treated PBMC-MΦ was demonstrated by Yamaguchi et al. These authors showed that activated TLR4 induced the release of elastase from neutrophils, which cleaved PAR2, thereby producing IL-12p40 (183). IL-12p40, a common subunit of IL-12 and IL-23, is involved in several pathogenic inflammatory responses associated with MΦ and dendritic cells (184). However, neutrophil elastase *per se* failed to increase IL-12p40 production in MΦ without PAR2 expression (183) (Figure 2A). Nakayama et al. showed that IL-32γ, a pro-inflammatory cytokine, also stimulated PAR2 signaling in a THP-1 macrophage cell line by generating proteinase-3 (PR3) (185). They showed that PR3 activated PAR2, which engaged with TRIF via the TIR domain to augment TNF-α and IFNγ generation. Because bacterial infection may cause endotoxin tolerance, the IL-32-PAR2-TRIF axis may act as an alternative signaling pathway to the LPS-TLR4-TRIF axis in shaping adaptive immunity (185).

However, Nhu et al. demonstrated that interaction between TLR4 and PAR2 may not be that simple. They showed that cooperative signaling between PAR2, TLR2, TLR3, and TLR4 induced NFκB activity to upregulate IL-8 expression, a gene principally involved in neutrophil chemotaxis. Additionally, the activation of PAR2 by PAR2-AP reduced TLR3-mediated STAT1 activation and TLR3/IRF3-induced IFNβ expression. However, for optimal PAR2 signaling, the presence of TLR4 was required. This cross-cooperativity was validated by the authors in an influenza-induced lethality mouse model. Here, the authors found that the Influenza A virus, which is known to activate the TLR3 pathway, did not produce any lethality in PAR2-null or TLR4-null mice, while significant lethality was noted in wild-type mice. This receptor cooperativity was also demonstrated in a PAR2-AP induced footpad edema model, in which PAR2-AP was not able to induce edema in TLR4 null or PAR2 null mice (174, 186).

In contrast to the above studies, Rayees et al., by performing bone marrow transplantation and adoptive transfer of macrophages, showed that PAR2 expressed in AMΦ counteracted the TLR4-induced inflammatory response by modulating Ca^2+^ entry and cAMP generation (31) (Figure 2B). It is known that Ca^2+^ entry induces the activities of both NFκB as well as the transcription factor NFAT, but in a cell-context dependent manner (187, 188). NFAT is basally phosphorylated, but when dephosphorylated by calcineurin, a Ca^2+^-dependent phosphatase, NFAT's transcriptional activity is turned on (188). Whereas, NFAT activity is known to regulate gene transcription in T cells, its role in MΦ remains understudied. Rayees et al. showed that PAR2 suppressed LPS-induced dephosphorylation of NFAT, i.e., activation of NFAT (31). These authors also showed mechanistically that PAR2 was required to suppress NFκB activity in part by blocking activation of NFAT. Thus, addition of 8-Br-cAMP, a membrane permeable cAMP-dependent protein kinase agonist, bypassed the requirement for PAR2 in diminishing TRPV4 activity and LPS-induced NFAT and NFκB activities as well as pro-inflammatory cytokine generation. These results, along with the findings listed above, identified the PAR2-cAMP cascade as a suppressor of TRPV4 activity and NFAT-mediated cytokine generation, thus demonstrating that thrombin activation of PAR2 in AMΦ blocks TLR4-mediated inflammatory signaling to reinstate tissue integrity (Figure 2B) (31). Nhu et al. similarly showed that simultaneous activation of PAR2 and TLR4, by PAR2-AP and LPS respectively, led to a decrease in expression of TNF-α, IL-6 and IL-12 in peritoneal MΦ, and enhanced expression of IL-10 (Figure 2B). These results were confirmed in LPS-treated PAR2-null MΦ, which showed significantly decreased IL-10 expression and, interestingly, the expression of CXCL1/KC, a strong neutrophil chemokine, was increased (174). Further studies using macrophage specific PAR2-null mice are required to resolve the role PAR2 plays in regulating TLR4 signaling. Also, a fundamental question that remains to be answered is whether exaggerated coupling of PAR1-mediated signaling with TLR4 in AMΦ is responsible for altering inflammatory injury in PAR2-null mice, as discussed above.

## Concluding Remarks

This review describes recent mechanistic developments in lung MΦ regulation of tissue-fluid homeostasis with an emphasis on PAR2-mediated signaling in AMΦ and its intersection with TLR4 signaling to modulate inflammation and lung vascular injury. We highlighted the subsets of lung resident MΦ and their dichotomous phenotypes, as regulated *in vitro* vs. *in vivo*. We also described recent advances in TLR4 signaling, such as the role of inflammasomes and STING in regulating AMΦ functions. We noted herein that generation of cAMP through PAR2 activity is critical in suppressing NFAT activity, thereby dampening AMΦ inflammatory signaling. Intriguingly, data also show the key role of cAMP in blocking TRPV4 activity in MΦ. However, several questions remain to be addressed: as for example

1. Where does this cAMP comes from to bind TRPV4 in AMΦ and how does cAMP alter the affinity of TRPV4 for its agonists?
2. Does PAR1 expression in AMΦ augment TLR4 activity in the absence of PAR2 expression?
3. How does PAR2, or PAR1 expression, for that matter in IMΦ or monocyte-derived MΦ, which are known to be recruited to the lung during injury, regulate AMΦ inflammatory signaling?
4. Are cAMP-induced transcription factors, such as CREB, involved in dictating AMΦ function? Further studies employing state of the art techniques such as macrophage imaging *in vivo* along with genetic mouse models will likely advance our understanding of lung MΦ subsets generation and function under normal conditions and during inflammation.

## Author Contributions

SR and DM: conceptualized the manuscript. IR and DM: edited the manuscript. SR, JJ, IR, BJ, SB, and DM wrote and reviewed the manuscript. All authors contributed to the article and approved the submitted version.

## Conflict of Interest

The authors declare that the research was conducted in the absence of any commercial or financial relationships that could be construed as a potential conflict of interest.
